# Platelet proteome changes in dogs with congestive heart failure

**DOI:** 10.1186/s12917-020-02692-x

**Published:** 2020-11-30

**Authors:** Pinar Levent, Meriç Kocaturk, Emel Akgun, Ahmet Saril, Ozge Cevik, Ahmet Tarik Baykal, Ryou Tanaka, Jose Joaquin Ceron, Zeki Yilmaz

**Affiliations:** 1 Department of Internal Medicine, Faculty of Veterinary Medicine, Bursa Uludag University, 16059 Bursa, Turkey; 2 Department of Medical Biochemistry, Acibadem University School of Medicine, Istanbul, Turkey; 3 Department of Basic Science, Medical Biochemistry, Adnan Menderes University School of Medicine, Aydin, Turkey; 4 Department of Veterinary Surgery, Faculty of Veterinary Medicine, Tokyo University of Agriculture and Technology, Tokyo, 183-8509 Japan; 5 Interdisciplinary Laboratory of Clinical Pathology, Interlab-UMU, University of Murcia, 30100 Murcia, Spain

**Keywords:** Platelet proteomic, Heart failure, Myxomatous mitral valve disease, Dogs

## Abstract

**Background:**

Platelets play a central role in the development of cardiovascular diseases and changes in their proteins are involved in the pathophysiology of heart diseases in humans. There is lack of knowledge about the possible role of platelets in congestive heart failure (CHF) in dogs. Thus, this study aimed to investigate the changes in global platelet proteomes in dogs with CHF, to clarify the possible role of platelets in the physiopathology of this disease. Healthy-dogs (*n* = 10) and dogs with acute CHF due to myxomatous mitral valve disease (MMVD, n = 10) were used. Acute CHF was defined based on the clinical (increased respiratory rate or difficulty breathing) and radiographic findings of pulmonary edema. Dogs Blood samples were collected into tubes with acid-citrate-dextrose, and platelet-pellets were obtained by centrifuge and washing steps. Platelet-proteomes were identified using LC-MS based label-free differential proteome expression analysis method and matched according to protein database for *Canis lupus familiaris*.

**Results:**

Totally 104 different proteins were identified in the platelets of the dogs being 4 out of them were significantly up-regulated and 6 down-regulated in acute CHF dogs. Guanine-nucleotide-binding protein, apolipoproteins (A-II and C-III) and clusterin levels increased, but CXC-motif-chemokine-10, cytochrome-C-oxidase-subunit-2, cathepsin-D, serine/threonine-protein-phosphatase-PP1-gamma-catalytic-subunit, creatine-kinase-B-type and myotrophin levels decreased in acute CHF dogs. These proteins are associated with several molecular functions, biological processes, signaling systems and immune-inflammatory responses.

**Conclusion:**

This study describes by first time the changes in the protein composition in platelets of dogs with acute CHF due to MMVD. Our findings provide a resource for increase the knowledge about the proteome of canine platelets and their roles in CHF caused by MMVD and could be a tool for further investigations about the prevention and treatment of this disease.

**Supplementary Information:**

The online version contains supplementary material available at 10.1186/s12917-020-02692-x.

## Background

Congestive heart failure (CHF) is one of the most common health problems in human [1, 2] and dogs [3]. In dogs, myxomatous mitral valve disease (MMVD) is a common cause of left-sided CHF. Mitral valve prolapse (MVP) characterized by abnormal systolic protrusion of the mitral valve leaflets into the left atrium due to progressive valve thickening is an important component of MMVD leading to CHF in these cases [3].

During the progression of CHF, turbulent high velocity blood flow and changes in blood shear stress around the degenerative mitral valve leaflets may produce platelet activation in dogs [4, 5]. Although clinical relevance of the platelet activation is not clear [6], some studies in this species have shown that activated platelets might contribute to the disease progression and an increased risk of sudden death by development of intra-myocardial coronary arteriosclerosis and micro-thrombi due to CHF [4–8]. Therefore platelets may play an important role in development and progression of cardiovascular diseases (CVDs) in dogs with or without overt-thromboembolism [7] as occurs in humans [9].

CVDs have comprised different pathology such as coronary artery disease (CAD), vascular diseases, heart valve disease, and cardiomyopathy, in which CAD is the most common in humans [9]. CAD defined as a chronic inflammatory disorder with a genetic susceptibility can remain stable for a long time, or induce the formation of an occlusive coronary thrombus followed by the cardiovascular complications such as myocardial infarction in humans [10]. MMVD represents the most common acquired CVD in dogs, and is characterized by slowly progressive valvular degeneration that causes mitral regurgitation and then cardiac remodeling and ventricular dysfunction [3]. Although both of them are related with the different etiopathogenesis, the common point of these pathologies seems to be platelets and platelet-related processes. Platelet proteins and their potential roles in the development of CVDs, especially in CAD, have been described in humans [2, 9, 10], but there is a lack of information about this subject in dogs with MMVD and/or CHF.

Platelets are the smallest blood cells produced from megakaryocytes in the bone marrow, and are involved not only in hemostasis, but also in several pathophysiological processes [11]. Since platelets contain about 5000 proteins, the importance and role of platelet proteins in the development and progression of diseases may be higher than expected [12]. With the latest technological advances, protein identifications from different samples by proteomic analysis can provide new details in the discrimination between diseased and healthy status. Modern platelet proteomic studies can reveal quantitative and post-transitional changes in proteins, protein-protein interactions and protein localizations [13]. Thus, platelet proteomics studies allow the characterization and elaboration of the basic biological, molecular and cellular functions of diseased and healthy conditions [14–16].

In humans, proteomic studies of platelets have been performed in CVDs [9, 10] and other pathological conditions such as sepsis, Alzheimer’s disease, diabetes mellitus, and uremia [2]. The alterations in the platelet-derived proteins such as adhesion molecules and the natriuretic peptide receptor were reported in humans with CHF [2]. Lower levels of haptoglobin and platelet basic protein were observed in plasma from humans with MVP as compared to the healthy controls [16]. MVP is not responsible per se for blood clotting activation, but the risk of thromboembolic events maybe increases in humans with severe mitral regurgitation [17]. Platelet coagulation hyperactivity was reported in patients with MVP and thromboembolism [18]. Platelet factor 4, a marker protein of platelet activation, was elevated in serum from MVP patients [19]. In a pacing-induced pig heart failure model, platelet proteins were involved in cellular processes ranging from proliferation to apoptosis, as well as inflammation and cytoskeletal changes, providing that signal transduction pathways in platelets might be key mediators of platelet contributions to cardiac failure [20]. Also, several platelet proteins such as amyloid A and apolipoprotein A1 indicated inflammatory involvement in the pathogenesis of heart failure in humans, while some of them such as cyclic nucleotide phosphodiesterase were associated with the protective role against β-adrenergic signaling and angiotensin II - induced cardiac remodeling in this disease [2]. These evidence shows that platelet proteins are involved in the pathophysiology of cardiovascular diseases in humans and experimental animals.

There are two studies reporting the platelet proteome in dogs, and both of them were performed in three healthy subjects [21, 22]. Cremer et al. [21] reported a total of 693 platelet proteins that were involved in coagulation, hemostasis, proteolysis, and organonitrogen compound metabolic process, and Trichler et al. [22] reported a total of 5.974 platelet proteins associated with various biological process such as response to stress, transport, cellular nitrogen compound metabolic process and signal transduction. However, with the exception of two studies in healthy dogs [21, 22], no platelet proteomic study has been performed in sick dogs. Thus, this study aimed to investigate the changes in global platelet proteomes in dogs with acute CHF due to MMVD, to clarify the possible role of platelets in the physiopathology of this disease.

## Results

### Clinical findings

Amongst the control dogs, five were female and five were male with different breeds (Labrador Retriever 3, Golden Retriever 3, Border Collie 2, Samoyed 1 and Anatolian shepherd 1), and ages (range 3.2 yrs. - 6.5 yrs). CHF group included 4 female and 6 male dogs, with different ages (3.5 yrs. – 13.0 yrs) and breeds (Golden Retriever 2, Labrador Retriever 2, Anatolian sheepdog 2, German shepherd 2, Cocker Spaniel 1 and mix-breed 1).

In this study, dogs with acute CHF (*n* = 10) suffered from exercise intolerance (10/10), respiratory distress and coughing (7/10) within 1 week before admission. Physical examination showed the presence of murmur over the mitral valve (10/10), and increased heart and respiratory rates in the patients. There were not statistically differences in body weight and age between groups (Table 1).

**Table 1:** Selected clinical, hematological and echocardiographic variables in healthy dogs and dogs with acute congestive health failure (CHF) (Mean ± Sd)

Parameter	Healthy Dogs *n* = 10	Dogs with acute CHF *n* = 10	*P* value
Clinical findings			
BW Kg	25.2 ± 6.5	22.4 ± 19.4	NS
Age years	5.3 ± 1.1	8.1 ± 5.1	NS
P bpm	111 ± 8	146 ± 28	< 0.001
R breath/min	27 ± 7	68 ± 21	< 0.001
VHS	9.3 ± 1.2	12.5 ± 1.5	< 0.001
Hematological and serum biochemistry findings			
Platelet ×10^3^/**μ**L	346 ± 66	325 ± 158	NS
cTnI ng/mL	0.04 ± 0.02	0.15 ± 0.1	< 0.05
Echocardiographic findings			
IVSDd cm	1.1 ± 0.4	0.7 ± 0.1	NS
LVDd cm	3.0 ± 0.7	5.0 ± 0.4	< 0.01
LVPWDd cm	1.2 ± 0.2	1.3 ± 0.4	NS
IVSSd cm	1.1 ± 0.3	1.1 ± 0.1	NS
LVSd cm	2.1 ± 0.5	3.2 ± 0.3	< 0.01
LVPWSd cm	1.1 ± 0.1	1.2 ± 0.3	NS
FS %	34.4 ± 3.8	26.0 ± 6.4	< 0.05
EF %	61.6 ± 9.2	48.0 ± 9.7	< 0.05
EPSS cm	0.3 ± 0.1	0.8 ± 0.1	< 0.001
LA/Ao	0.9 ± 0.2	2.6 ± 0.9	< 0.001
PV/PA	0.9 ± 0.3	2.6 ± 0.4	< 0.001
LVIDDn	1.16 ± 0.1	2.01 ± 0.2	< 0.01
MV E/A	1.5 ± 0.3	3.8 ± 1.5	< 0.05

*BW* body weight, *P* pulsation, *R* respiration, *VHS* vertebral heart score, *cTnI* cardiac troponin I, *IVSDd* interventricular septum diastole diameter, *IVSSd* interventricular septum systole diameter, *LVDd* left ventricular diastole diameter, *LVSd* left ventricular systole diameter, *LVPWDd* left ventricular post wall diastole diameter, *LVPWSd* left ventricular post wall systole diameter, *FS* fractional shortening, *EF* ejection fraction, *EPSS E* point to septal separation, *LA/Ao* left atrium to aortic ratio, *PV/PA* pulmonary vein to pulmonary artery ratio, *LVIDDn* normalized left ventricular internal dimension in diastole, *MV E/A* mitral valve early ventricular (E) and late atrial contraction (A), *NS* statistically not significant

In dogs with acute CHF, thoracic radiography showed a left atrial enlargement (mild 3, and moderate - severe 7), pulmonary venous congestion (10/10), pulmonary infiltrates (mild interstitial 3, diffuse interstitial 4 and alveolar 3), pleural effusion (3/10), and intra-thoracic dorsal deviation of Vena cava and trachea (10/10). ECG examination revealed morphologically prolonged P wave duration (8/10), increased QRS voltage (7/10), and prolonged QRS duration (7/10), and rhythm analysis showed the presence of sinus tachycardia (*n* = 6) and atrial fibrillation (*n* = 4).

A significant increase in vertebral heart score was detected in acute CHF dogs compared to the controls. Compared to the control group, LVDd, LVDs, LVIDDn, and EPSS values were higher (*P* < 0.01), but FS%, EF%, and MV E/A values were lower (*P* < 0.05) in dogs with acute CHF. Pulmonary vein to pulmonary artery ratio (PV/PA) was higher in dogs with acute CHF than that of healthy controls (*P* < 0.001) (Table 1). Mitral regurgitant jet velocity varied from mild to moderate - severe. There were not statistically differences in maximal velocities of MPA and Ao Doppler flows between groups (data not shown).

### Platelet proteomic findings

**Fig. 1:**
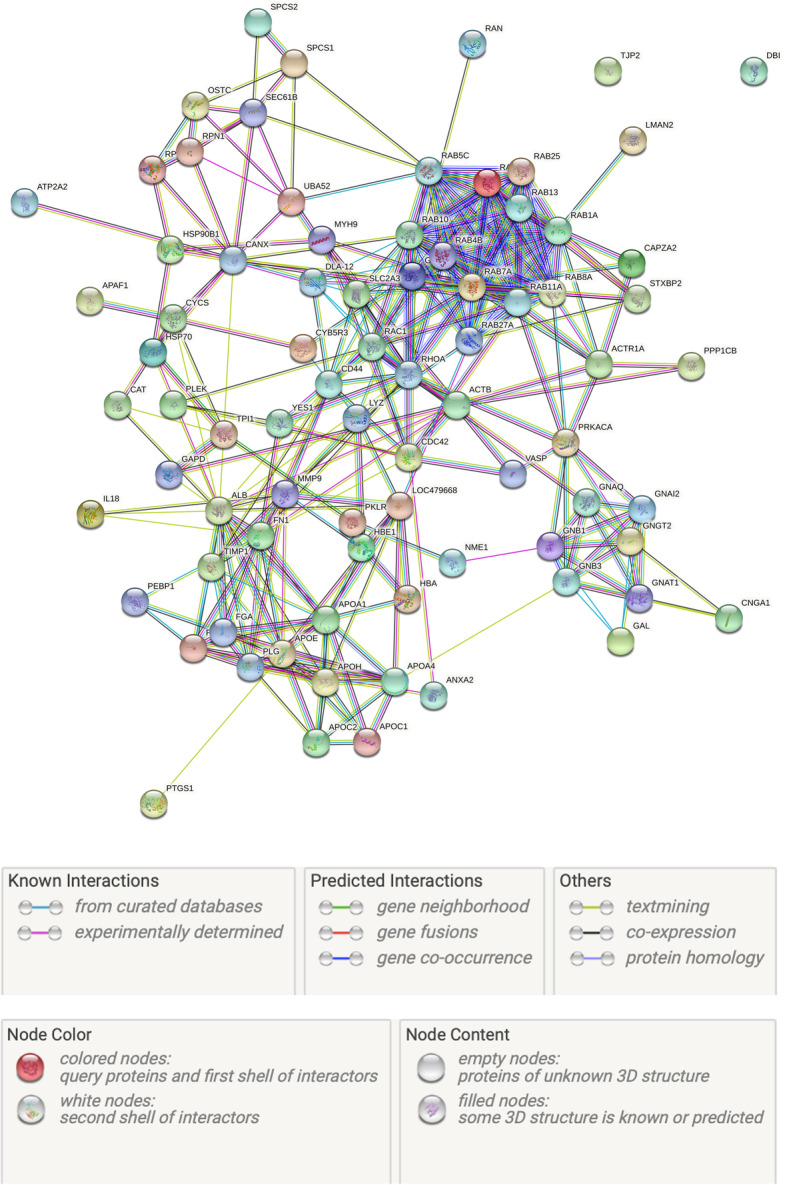
Interactions between all proteomes (*n* = 104) that were described for *Canis lupus familialis* according to protein gene bank. Protein-protein interaction showed a high interaction potential among COX enzymes, RAS-related proteins, Guanine nucleotide-binding protein (GNB) and apolipoproteins (APOA-II, APOC-III, and APOJ or clusterin) in dogs with acute CHF. Protein – protein interaction was showed by String analysis. Figure and legends were created by an open source server (www.string-db.org)

In the present study, a total of 104 common differentially expressed proteins were identified, and 10 out of them showed at least 1.2 fold-change at statistically significant level between two groups (Table 2). Compared with the healthy group, some proteins were increased (up-regulation, *n* = 4) and some proteins were decreased (down-regulation, *n* = 6) in dogs with acute CHF (Table 2). Platelet proteins; apolipoprotein A-II (ApoA-II), apolipoprotein C-III (ApoC-III), guanine nucleotide-binding protein (GBP) subunit alpha-11 and clusterin increased. On the other hand, cathepsin D, cytochrome C oxidase (COX) subunit 2, CXC motif chemokine 10 (CXCL10), serine/threonine - protein phosphatase PP1 - gamma catalytic subunit (PPP1CC), myotrophin and creatine kinase B-type (CKB) decreased in dogs with acute CHF. Other proteins (*n* = 94) that were detected in our study were given as a supplementary file (Additional file 1), and included RAS related proteins (RAB), Guanine nucleotide-binding protein (GNB), Apolipoproteins (APOs), cAMP-dependent protein kinase catalytic subunit alpha (PRKACA), Fibronectin (FN), Matrix metalloproteinase-9 (MMP-9), Metalloproteinase inhibitor 1 (TIMP1), Actin cytoplasmic 1 (ACT), Heat shock proteins (HSP), and haptogloblin (HP) among others (Fig. 1 and Additional file 1).

**Table 2:** Accession number, peptides, scores, fold-changes and description of the platelet proteomes (*n* = 10) that were differentially expressed in dogs with congestive heart failure compared to controls. Proteins are listed according to up or down regulation and fold change

Accession no	Peptides^a^	*P* value	Fold change	Protein description	Up or down
P12279	6 (5)	0.04	2,01	Apolipoprotein C-III	Up
P52206	19 (10)	0.02	2,07	Guanine nucleotide-binding protein subunit alpha-11 (Fragment)	Up
E2RAK7	16 (14)	0.03	1,94	Apolipoprotein A-II	Up
P25473	68 (59)	0.03	1,81	Clusterin	Up
Q5KSV9	1 (1)	0.02	2,87	C-X-C motif chemokine 10	Down
P67780	6 (5)	0.02	1,49	Cytochrome C oxidase subunit 2	Down
Q4LAL9	6 (6)	0.04	1,46	Cathepsin D	Down
P05124	51 (45)	0.04	1,28	Creatine kinase B-type	Down
Q8MJ46	13 (6)	0.03	1,24	Serine/threonine-protein phosphatase PP1-gamma catalytic sub.	Down
Q863Z4	19 (16)	0.04	1,20	Myotrophin	Down

^a^ Peptide column shows two values where there first number is the number of identified peptides and the second number in the parentheses is the number of non-conflicting peptides included in intensity calculations

Protein identifications are done against the reviewed *Canis lupus familiaris* protein database from https://www.uniprot.org/

String analysis of the 10 proteins that significant changes in their expression in CHF showed a relationship between the group of COX proteins (COX-I, COX-II and COX-III) and cytochrome B oxidase (CYTB) with unspecified action of binding and catalysis. CXCL10 stimulates positively G protein -coupled receptor (CXCR3). There were also interactions between apolipoproteins (ApoA-II, ApoC-III and ApoJ or clusterin) in dogs with acute CHF (Fig. 1). There were interactions between some significant proteins and others listed in 97 proteins, such as catalytic activity between PPP1CC and RAB1B, post-translational modification between GNB and PRKACA, and binding and catalytic activity GNB and CXCL-10 (Fig. 1).

Panther Go-Slim analysis showed the protein classifications based on biological process, cellular component, molecular function and protein class for all identified proteins (Additional file 2 that included 5 supplementary figures). The role of proteins showing statistically significant changes in abundance between groups included biological processes such as biological regulation (GNB, CXCL10 and apo A-II), biogenesis (Apo-AII), signaling (GNB and CXCL10) and cellular (COX, CK B-type, GNB, CXCL10 and apo A-II) and immune system processes (CXCL10), molecular functions such as binding (GNB, CXCL10, clusterin and ApoA-II), catalytic activity (COX, CK B-type, GNB, PPP1CC and ApoA-II), molecular function regulator (CXCL10 and apo A-II) and transporter activity (COX). These proteins were of cell part (GNB, cathepsin D, clusterin and PPP1CC), extracellular region (CK B-type, CXCL10, clusterin and apo-AII), macromolecular complex (GNB and Apo-AII), membrane (GNB) and organelle (cathepsin, clusterin and PPP1CC). According to Panther pathway analysis, these proteins have a role in several signaling systems such as chemokine and cytokine mediated inflammatory pathway (GNB and CXCL10), alpha-adrenergic and endothelin signaling pathways (GNB), G-protein signaling pathway (GNB), dopamine receptor mediated signaling pathway (PPP1CC), and Wnt signaling pathway (GNB).

**Fig. 2:**
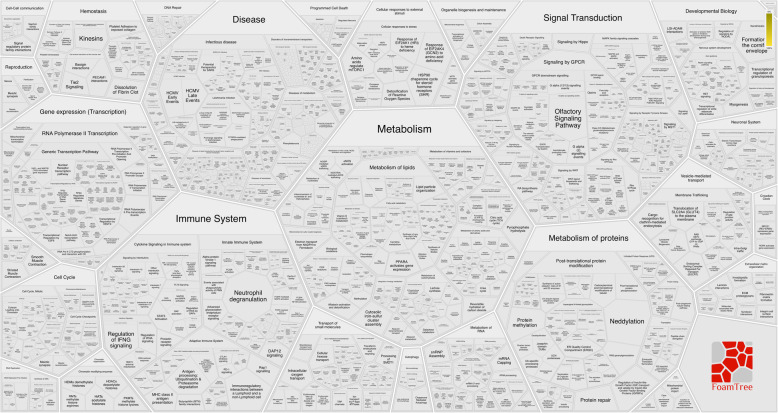
Reactome analysis and its figure were performed by an open source server (www.reactome.org). This analysis suggested that metabolism, immune system, cell cycle, gene expression (transcription), signal transduction, and hemostasis may be critical in the development of acute congestive heart failure (CHF) in dogs

In the Reactome analysis, 10 proteins were found to be associated with metabolism, immune system, cell cycle, gene expression (transcription), signal transduction, and hemostasis (their details were given in Fig. 2).

## Discussion

In this study, changes in proteins of the platelets are described in dogs with acute CHF and MMVD. This represents to the author’s knowledge the first report in which changes in the proteins of platelets are reported in dogs with any disease. Platelet proteins may have a role in the pathophysiology of CHF due to MMVD by regulating several molecular functions, biological processes and signaling systems. Therefore, this study could be a basis for future developments in order to better elucidate the role of platelets in the physiopathology of acute CHF and other different diseases in the dog, which could lead to new strategies of treatment and management of these conditions.

Acute CHF due to MMVD in dogs studied were diagnosed based on current clinical (tachypnea and respiratory stress, etc.), radiographic (increased radiographic composite score) and echocardiographic findings (left-sided cardiac remodeling and mitral valve degeneration, etc.), and classified as stage C according to ACVIM heart failure guidelines for dogs [3]. The reason why stage C was selected in this study is because it corresponds to symptomatic dogs but without receiving any medication [3]. Thereby the possible effects of cardiac drugs (inotropes, diuretics, beta-blockers and ACE inhibitors) on platelet functions were avoided [11].

In this study, a total of 104 platelet proteins were identified, in which 10 were found to be differentially expressed at statistically significant level. Some of the proteins (ApoA-II, GBP, cathepsin D, COX2, CXCL10, PPP1 and CKB) were already identified from healthy dogs in a previous study [22], whereas some proteins such as myotrophin and ApoC-III are described herein by first time in the dog.

Platelet myotrophin activity was decreased in this study, in line with a previous report in humans with CHF in which this protein was decreased in plasma samples [23]. Myotrophin is hypertrophy-inducing factor, which acts in the formal arrangement of actin filaments and promotes cardiac muscle hypertrophy [23, 24], and was reported as a serum biomarker showing early activation in CHF [23]. Additionally, myotrophin stimulates the transcription factor-kappa B (NF-kB) activity which regulates expression of several genes involved in immune responses, inflammation, proliferation, and apoptosis in ventricular myocytes. Therefore, decreasing platelet myotrophin activity in this study may be a protective response in the pathophysiology of CHF by limiting the NF-kB activity on cardiomyocytes [25], and the initiation process of myocardial hypertrophy in response to volume overload [26].

We have identified three apolipoproteins in this study; ApoC-III, ApoA-II, and clusterin (ApoJ). ApoC-III is a regulator of triglyceride rich lipoproteins which leads to hypertriglyceridemia and then cardiovascular disease [27–30]. In line with our results, Roura et al. [30] found a significant increase in serum ApoC-III in people with dilated cardiomyopathy compared to controls. ApoC-III was described as a thrombogenic factor for cardiac patients due to complex interactions with plasma endogenous thrombin formation and coagulation cascade [27], as well as an increase in the accumulation of atherogenic lipoproteins in the vessel wall [29]. Therefore, Apo-CIII could alter the homeostatic balance in a pro-coagulant way, and may promote atherothrombotic complications in dogs with CHF [31]. Although these complications cannot be detected clinically during diagnostic work-up in dogs, it should be kept in mind that increasing Apo-CIII may be a risk factor for development of intra-myocardial coronary arteriosclerosis and micro-thrombi which were reported in dogs with CHF [4, 7, 8].

ApoA-II is a major component of HDL particles [27, 28], and controls reverse cholesterol transport to inhibit the development of atherosclerosis [32, 33]. Antithrombotic effects of ApoA-II and HDL complex were related to inhibition of the coagulation cascade by stimulating endothelial production of nitric oxide and prostaglandins, and stimulation of clot fibrinolysis [34]. Low level of ApoA-II was associated with increased severity and worse outcomes in heart failure patients [35]. Thus, in this study, increasing platelet ApoA-II levels in dogs may be considered as a host response to be prevented from thrombotic effects and to manage fibrinolytic mechanisms during CHF.

Serum clusterin levels were reported to increase in various conditions such as myocardial infarction, inflammation, apoptosis, and oxidative stress [36, 37]. Overall it has a protective effect by preventing endothelial activation (anti-atherosclerotic effect) [36–38] and alleviating angiotensin II - mediated damage of cardiomyocytes, a key mechanism in the pathogenesis and progression of CHF [39]. In the present study, increased level of platelet clusterin may be a cytoprotective reaction against progression of acute CHF. In contrast, that serum clusterin level decreased due to continuous consumption was associated with an unfavorable prognosis in patients with chronic heart failure [37].

GBP subunit alpha-11, called as “G protein”, plays a central physiological role in the regulation of cardiac contractility by neurohumoral signals [40, 41]. In addition, G proteins modulate the binding of angiotensin-II to adrenal cortex receptors in the homeostatic regulation of the cardiovascular system [42, 43]. The increase found in this study agrees with previous reports in humans with cardiomyopathy, where functional activity of G protein increased [44]. Upregulation of GNB in dogs with CHF may be considered as an adaptive protective response after myocardial injury (evidenced by increased serum cTnI levels) to prevent myocytes from apoptosis as reported in mice with ischemic stress [45], and regulate cardiac contractility during CHF.

CXCL10, also known as interferon-inducible protein-10 (IP-10), a member of chemokine family, was one of the down-regulated platelet proteins in dogs with acute CHF in the present study, similar to the results of serum CXCL10 in mice with myocardial infarction [46]. After releasing from leukocytes and endothelial cells, CXCL10 binds to its receptor (G protein – coupled receptor; CXCR3), and leads to a range of inflammatory and immune responses (meaning the regulation of leukocyte and lymphocyte traffic to the damaged tissue); key factors in cardiovascular diseases (CVD) such as atherosclerosis, myocardial infarction [46–48], and cardiac remodeling [49]. Circulating CXCL10 was found as the best indicators amongst others chemokines for differentiating healthy and heart failure patients [49].

COX enzyme plays important role for mitochondrial oxidative metabolism and ATP synthesis. In this study, COX-II was one of the down-regulated platelet proteins in dogs with acute CHF. COX functions are affected by several pathological conditions including myocardial ischemia [50] and cardiomyopathy in humans [51]. COX deficiency was associated with increased mitochondrial reactive oxygen species (ROS) production and cellular toxicity [50]. Increasing oxidative stress lead to pathological changes in COX structure and function, resulting in exacerbating apoptosis in patients with CHF [52]. In the light of this information, down-regulated COX-II protein may be resulted from its excessive use in response to increased oxidative stress in the progression of CHF in dogs. Since COX enzyme handles more than 90% of molecular oxygen produced by the mammalian cells and tissue [50], low COX-II levels may be a factor in the emergence of exercise intolerance and/or respiratory stress in dogs with acute CHF, as reported in humans [52]. Platelet COX activity enhancing drugs may have a potential to limit the progression of adverse cardiac remodeling and heart failure, as suggested for septic patients with low COX activity [53].

Similarly to our study, serum levels of cathepsin D were down-regulated in human with heart failure [54] and myocardial infarction [55]. Cathepsin D plays a role in cardiomyocyte autophagy, which protects against the progression of post-infarction cardiac remodeling [55]. In this study, decreased cathepsin D was most probably resulted from its excessive use for myocardial damage contributing to left sided cardiac remodeling in dogs. This may reflect the impaired protective role of cathepsin D [55], meaning less autophagy in these dogs.

Finally, there were other two proteins showing downregulation in acute CHF dogs, the cytosolic brain type homodimeric - creatine kinase (CKB) and type 1 of serine/threonine phosphatases (PP1). The lower CKB concentrations in dogs with acute CHF may be contribute to contractile dysfunction resulted from impaired myocardial energy metabolism in these patients [56]. PP1 is considered a key regulator of cardiac function, and modulation of its activity may represent a novel therapeutic target in heart failure in humans [57, 58] and dogs.

Protein-protein interaction (string analysis) showed a high interaction potential among COX enzymes in dogs with acute CHF. Interactions between COX enzymes have been described in a monkey model of heart failure leading to increased apoptosis in cardiomyocytes [52]. Also, we observed an interaction by unspecified catalytic and binding activity between COX and APAF-1 (apoptotic protease activating factor-1), this could an additional cause of apoptosis playing a central role in the loss of cardiomyocytes in patients with cardiomyopathy, leading to a progressive decline in left ventricular function, and CHF [59, 60].

CXCL10 stimulates positively G protein-coupled receptor (CXCR3) and CXCR3 is a chemokine receptor that is expressed mainly on effector T cells [61]. Therefore, both of them may play an important role in immune-inflammatory reaction such as T cell trafficking and function during CHF in dogs. Interaction between them activates a number of signaling pathways such as MAP kinases [62] and PI3K/Akt signaling involved in the pathophysiology of heart failure [63].

Interactions between different apolipoproteins have also described in the pathogenesis of human CHF [64]. All proteins that showed significant changes in this study seemed to have interactions by one or more chemical pathways. This indicated the complexity of the pathogenesis of heart failure and further molecular studies are needed to explain these interactions in the pathogenesis of CHF.

In the Reactome analysis, 10 proteins that showed significant changes were found to be associated with various functions such as hemostasis, signal transduction, cell cycle, gene expression (transcription), immune system, protein metabolism, muscle contraction, apoptosis, and extracellular matrix and chromatin organizations.

Panther pathway analysis of observed platelet proteins support further the roles of chemokine and cytokine mediated inflammatory pathway, and alpha-adrenergic and endothelin signaling pathways in involvement of the pathogenesis of acute CHF, in agreement with the previous studies of an experimental model of heart failure in swine [20], and humans with heart failure [2]. Another activated pathways such as gonadotropin-releasing hormone receptor pathway and Wnt signaling pathway might be associated with cardiac contractile function and cardiac remodeling in dogs, as reported in humans [65, 66].

There are some limitations for this study. The first is that there is no validation study of the proteomics results by using other techniques such as Western blot in a larger number of samples [67]. Therefore this study should be considered as a pilot study and further studies for validate our findings should be made. Second, if the dogs studied here suffered from CAD remained unclear. Platelet or serum proteomic studies are performed in humans with CVDs, such as CAD [10] and atherosclerosis in humans [9, 27, 28, 32, 46], whereas this study was carried out in dogs with MMVD and CHF. We could not examine the dogs if they had suffered from CAD, by cardiac catheterization and angiogram and cardiac CT scan during diagnostic work-up. In a previous study, histopathologically confirmed intramural atherosclerosis was reported in dogs with acute and chronic CHF [68], suggesting the possibility of atherosclerotic changes during CHF in dogs. Third, there are wide ranges of ages within groups, and a difference (but not statistically significant) between groups. Platelets of aged subjects show some differences compared to those of young peoples in several aspects such as lower aggregation capacity, higher levels of β-thromboglobulin and platelet factor 4, and decreased levels of prostacyclin [69]. In addition, age-related changes in platelet function seem more profound in women than in men indicating that age and gender can affect significantly on platelet functions [70]. Therefore, additional studies are needed to elucidate the connection between the changes in platelet proteins and age and gender differences in dogs with healthy and various pathological conditions [71].

## Conclusions

In conclusion, in dogs with acute CHF due to MMVD there are changes in the composition of the proteins of platelets. The proteins that change are associated to cellular, biologic, metabolic, immune, and coagulation system processes involved in the development of acute CHF caused by MMVD. These proteins could be potential biomarkers and also targets for the development of new therapeutic and prophylactic strategies in dogs with heart failure.

## Methods

This prospective study was performed between June 2017 and September 2018 at the Veterinary Teaching Hospital, Bursa Uludag University (BUU), Bursa / Turkey.

### Dogs and groups

This study consisted of a total of 20 client-owned dogs of different breed, age, body weight, and both sexes. The dogs were divided in two groups: a control group integrated by healthy dogs (*n* = 10) and the group of dogs with acute CHF (n = 10). A complete physical, laboratory, thoracic radiography, electrocardiography (ECG) and echocardiographic examination were made in order to include each dog in the group of healthy and acute CHF dogs.

### Inclusion criteria

This study included only dogs with stage C acute CHF due to MMVD. CHF was staged according to the American College of Veterinary Internal Medicine (ACVIM) consensus guidelines for the diagnosis and treatment of MMVD in dogs [3]. For this study, dogs were selected, showing both criteria at the same time: 1) presence of clinical signs of CHF (coughing, exercise intolerance, and tachypnea, etc.) along with the presence of physical examination findings such as systolic heart murmur (at least grade 4/6 over the mitral puncta maxima) and precordial thrill, and 2) radiographic (vertebral heart score [VHS] > 11.0) and echocardiographic evidence (left atrial to aortic root ratio [LA/Ao] > 1.6 and/or left ventricular end diastolic diameter normalized for body weight [LVIDDn] > 1.7) of left-sided cardiac remodeling due to MMVD [3, 72].

MMVD was diagnosed by trans-thoracic echocardiography (CarisPlus®, color Doppler, Italy), based on the combination of following criteria: the presence of mitral valve prolapse (MVP) and/or thickening of the mitral valve leaflets by 2-D echocardiography on right parasternal long-axis (RPLA) and apical 4-chamber views, and identification of mitral valve regurgitation on left apical 4-chamber view by color Doppler examination. Mitral regurgitation was also confirmed by color M-mode examination at right parasternal short axis view – mitral valve level [73, 74].

The cases of acute CHF due to MMVD were selected, and acute CHF was characterized by the current clinical (increased respiratory rate or difficulty breathing) and radiographic findings of pulmonary edema. For this purpose, thoracic radiographic score was used to describe the probability of CHF. Briefly, this score was estimated based on the absence or presence (and its severity) of left atrial enlargement, pulmonary venous congestion, pulmonary infiltrates, and pleural effusion [75]. The dogs with score > 4 were forwarded to echocardiographic examination for final diagnosis. Radiographic and clinical findings were interpreted in a combination with the echocardiography, providing a final diagnosis of acute CHF due to MMVD [73–75].

In addition, these dogs had at least one cardiac rhythm abnormalities (i.e., sinus tachycardia and atrial fibrillation) identified by ECG and higher serum cardiac troponin I (cTnI) level than the reference ranges (< 0.03–0.07 ng/mL) at the time of first admission to the clinic (I-STAT®, Abaxis). Cardiologic examination was performed by PL (first author) and then MMVD with Stage C CHF was confirmed by specialists, MK and ZY.

Control dogs were obtained from staff and students of BUU Veterinary Teaching Hospital. These control dogs according to the owners did not have any history of chronic disease and in the last 2 months did have any disease and not receive any drug, preventive treatment or vaccination. These dogs did not show any alteration at physical and cardiological examination (including ECG and echocardiography) and showed values of hemogram and biochemistry analysis (including serum cTnI) within the reference values of our laboratory.

### Exclusion criteria

Cases with a history of any disease different to MMVD and CHF for the past 2 months and that had any chronic disease or were under medication were excluded of the study. Dogs were excluded if they had a congenital heart disease or another acquired cardiac disorder such as dilated cardiomyopathy (DCM). Asymptomatic dogs with MMVD with (stage B2) or without cardiac remodeling (stage B1) and symptomatic dogs with refractory CHF (stage D) were also excluded. The dogs who had a lower than or equal to 4 of radiographic composite score were not included in this study. Besides, routine hematological and biochemical data were used to identify and exclude patients who had anemia, renal failure, endocrine diseases (diabetes mellitus and hypothyroidism), or hepatobiliary diseases accompanying primary problem. Ehrlichia, Lyme disease, Dirofilaria, Leishmania and/or Anaplasma positive cases were excluded by rapid screening tests (Anigen CaniV and LeishVet, Bionate). If the dogs received the medication such as steroids, non-steroids, antibiotics, inotropes or diuretics before inclusion, they were not included to the study, because of their potential effects on platelet protein expressions.

The dogs included in the study were treated by conventional medical approaches (diuretics, inotropic, and angiotensin converting enzyme inhibitors, etc.) for heart failure, and then re-examined as needed. Thus, dogs studied continued their lives under the responsibility of the patient owners. We have a permission to collect the animals’ samples from the owners.

### Sample collection and measurements

#### Clinical examinations

In this study, cardiological examination was performed following the well-known procedures including physical examination, electrocardiography (ECG), thoracic radiography and echocardiography on admission day in all dogs. Thoracic radiographs were taken in different imaging planes (right and left lateral, and ventrodorsal positions) to evaluate the heart, vertebral heart score, lung and thoracic vessels, and then each of which was used to estimate radiographic composite score [75].

ECG was performed using 3 bipolar standard limb leads in un-sedated dogs. Cardiac rhythm analyses and measurements were performed with a standard calibration (10 mm/mV and 50 mm/sec), as reported in our previous studies [76, 77].

Echocardiographic examinations were performed using conventional methods (2-D, M-mode, and color Doppler) and imaging techniques (right parasternal short – RPSA, and long axis - RPLA, left apical 4–5 chamber and subcostal views) with three different choices (2.5–5, 5–7.5, and 7.5–10 MHz) of phased-array cardiac transducers in all dogs (Caris Plus Esaote, Italy) [76–78]. Left ventricular (LV) - related parameters such as interventricular septum (IVS) and post-wall (LVPW) thickness, LV internal dimension at diastole and systole (LVDd and LVDs), and E-point to septal separation (EPSS) were measured at RPLA 4 chamber view by 2D and M-mode echocardiography. LV systolic function was evaluated by ejection fraction (EF %) and fractional-shortening (FS %), and both of them were calculated automatically with M-mode measurement (Teicholz method). The LA/Ao was obtained from the 2-D RPSA view – aortic level. LVDDn was calculated according to Cornell’s method of allometric scaling: LVDDn = LVDd (cm) /body weight (kg)^0.294^ [72]. For PV/PA estimation, PV and PA diameters were measured by M-Mode echo at RPSLA 4-chamber view as previously described [79].

LV diastolic function was evaluated by trans-mitral inflow profile including early LV filling (E), late atrial contraction (A) and their ratio (E/A) by Pulsed-wave Doppler echo at apical 4-chamber view. At the same image, the presence of mitral regurgitation was determined by color Doppler mode, and its severity was estimated by the measurements of regurgitant jet velocity with continuous Doppler mode [80]. As a standard procedure, other measurements such as main pulmonary artery (MPA), and Ao Doppler flow velocities were determined as suggested [81].

#### Laboratory examinations

Blood samples were collected, at the same day of cardiological and radiographic examinations, before starting to treatment, from the cephalic veins into EDTA containing tubes for complete blood counting, and acid citrate dextrose (ACD) containing tubes for platelet isolation [15], as well as into anticoagulant-free tubes for routine serum biochemistry analysis.

For platelet isolation, a volume of 20 ml of venous blood was collected into the tubes with ACD containing trisodium citrate (22.0 g/L), citric acid (8.0 g/L) and dextrose (24.5 g/L) (BD Vacutainer), and platelets were then isolated according to the method modified from Cevik et al. [15]. Briefly, platelet isolation steps included:

i- Blood was drawn into ACD tubes for platelet isolation.
ii- It was centrifuged (150 g / 15 min, at room temperature) to obtain platelet-rich plasma (PRP).
iii- PRP was transferred to dry tubes. HEPES buffer solution (137 mM NaCl, 3.8 mM HEPES, 5.6 mM Glucose, 2.7 mM KCl, 1 mM Magnesium sulfate, pH 7.4; Sigma) was added over the PRP samples. This was suspended with prostaglandin E1 (1 μM/L, Sigma) to inhibit platelet activation during high-speed centrifugation [82]. This mix was centrifuged at 800 g for 15 min to concentrate the platelets.
iv- Platelet pellets were re-suspended in ammonium bicarbonate solution (50 mM, Sigma) and then centrifuged at 800 g for 15 min to obtain final platelet pellets. Platelet extractions with WBCs and RBCs contaminations less than 0.5 and 0.1% by Diff-Quick staining, respectively, were considered sufficient to perform platelet proteomic study.

Serum samples and pure platelet pellets were stored in cryo tubes at − 80 degrees. After the sample collections in both groups were completed, all platelet pellets were analysed at the same time.

#### Proteomic analysis

Label-free differential proteomics analysis method was used to detect altered protein expressions in this study. The method provides changes in protein amount across the sample set rather than quantifying absolute amounts of each protein in the samples. Protein quantitation is based on the ion intensities of its non-conflicting peptide features. Unique peptides identified for the protein is used for calculating the protein intensity. Differential expression changes of the proteins reported here are relative ratios showing the up-regulation or down-regulation of a specific protein.

LC-MS based label free proteomics analysis was done for 10 control samples and 10 CHF samples being each sample individually analysed. Every injected sample is a separate biological replicate. Steps of platelet proteomic analysis were performed in a total of 20 different biological samples, as described in a previous paper [15]. Before starting the analysis, the detector and calibration settings were made by the MassLynx program (V4.1-Waters) which is specific to Xevo G2-XS Q TOF (Waters) device where the analysis were performed. The method was switched to SONAR and sensitivity mode and the tryptic peptides generated were subjected to 132 min reverse phase chromatography at 300 nL / min flow rate in a HSS T3 (Waters-186,008,818) nano column. Separation of the peptides from the column was achieved by increasing in the range of 5–35% acetonitrile according to their hydrophobicity and analyzed by mass spectrometry. During the analysis, data were collected for peptides that could be identified in the m / z range 50–1950. MS analysis was performed for 0.7 s and information was collected about the entire peptide. Then, MS / MS analysis was performed for 0.7 s and the peptide fragmentation and sequence information were obtained.

Throughout this analyses, [Glu1]-fibrinopeptide B human peptide standard was infused to the mass spectrometer every 60 s in a 50:50 ACN:Water mixture to be used as a mass calibrant. Peptides were mass re-calibrated based on the standard peptide mass value. In a label free data independent acquisition method an intensity calibrant was not used. Our results are relative ratios and not absolute quantities. All data pertaining to protein identifications with peptide sequences, mass errors and modifications are given as a supplementary file (Additional file 3).

### Statistical analysis

In this study, the *student t* test was applied for the clinical, laboratory (hematologic and biochemical) and cardiological examination results in two groups. Results were given as mean ± standard deviation, and *P* < 0.05 was considered statistically significant (SigmaStat 12.0, GmBH). Proteins obtained in three separate sets were paired with previously described proteins for *Canis lupus familiaris* in the gene bank. Protein identification and statistical analysis was performed using Progenesis QIP software (Waters-2018). In the method, samples from different patients were compared as a group and the proteins separating the groups were identified. Various statistical calculations were used in order to test reliability of the results obtained, to compare hundreds of proteins at the same time and to reach significant information by software package programs. Platelet proteins with at least P < 0.05 and more than 1.2-fold change were accepted as significant in dogs with acute CHF due to MMVD compared to control group.

Bioinformatic analysis was performed to show the protein – protein interaction (STRING; www.string-db.org), and the roles of proteins in molecular, cellular, and biological process, and pathway analysis was made to evaluate the possible association between proteins and biological functions (REACTOME; www.reactome.org and www.pantherdb.org).

## Supplementary Information

**Additional file 1 **Accession number, fold changes and description of the platelet proteomes (*n* = 94). This is a supplementary file including that 94 out of 104 platelet proteins were differentially expressed but not statistically significant in dogs with heart failure compared to controls.

**Additional file 2.** Protein classification. Protein classification based on the biological process, molecular function, protein class and pathway analysis.

**Additional file 3.** Protein identifications and analytic details. Protein identifications with peptide sequences, mass errors and modifications.

## Data Availability

All data in this study will be available from the corresponding author upon reasonable previous request and with the permission of the research fund. Protein identifications are done against the reviewed *Canis lupus familiaris* protein database from https://www.uniprot.org/. All data pertaining to protein identifications with peptide sequences, mass errors and modifications are given as a supplementary file (Additional file 3).

## Abbreviations

ACVIM: American College of Veterinary Internal Medicine
ApoA-II: Apolipoproteins A-II
ApoC-III: Aopoliporotein C-III
CHF: Chronic heart failure
CKB: Creatine kinase type B
COX: Cytochrome-C-oxidase
cTnI: cardiac troponin I
CXCL10: CXC-motif chemokine-10
GBP: Guanine nucleotide-binding protein
HDL: High-density lipoprotein
ROS: Reactive oxygen species
MMVD: Myxomatous mitral valve degeneration
PP1: Serine/threonine-protein phosphatase PP1-gamma catalytic subunit
