# Supplementary material for: Platelet proteome changes in dogs with congestive heart failure
Source: BMC Vet Res. 2020 Nov 30;16:466. doi: 10.1186/s12917-020-02692-x (PMC7708215; doi:10.1186/s12917-020-02692-x)
Supplement: Supplementary file 1 — Additional file 1 Accession number, fold changes and description of the platelet proteomes (n = 94). This is a supplementary file including that 94 out of 104 platelet proteins were differentially expressed but not statistically significant in dogs with heart failure compared to controls. [file 12917_2020_2692_MOESM1_ESM.docx]

**Supplementary data:**

**Additional file 1:** Accession number, fold changes and description of the platelet proteomes that were differentially expressed but not statistically significant (P>0.05) in dogs with heart failure compared to controls.

Protein identifications are done against the reviewed *Canis lupus familiaris* protein database from [https://www.uniprot.org/](https://www.uniprot.org/).

| **Number** | **Fold Change** | **Protein description** |
| --- | --- | --- |
| **P41148** | 1,1 | Endoplasmin |
| **P50551** | 1,1 | Vasodilator-stimulated phosphoprotein |
| **O46674** | 1,2 | Sarcoplasmic/endoplasmic reticulum calcium ATPase 2 |
| **Q8MJ47** | 1,3 | Serine/threonine-protein phosphatase PP1-beta catalytic subunit |
| **P60524** | 1,8 | Hemoglobin subunit beta |
| **P81709** | 4,4 | Lysozyme C-spleen isozyme |
| **P24409** | 1,5 | Ras-related protein Rab-10 |
| **P49822** | 3,2 | Serum albumin |
| **Q8HZR1** | 1,8 | Prostaglandin G/H synthase 1 |
| **Q28279** | 1,2 | cGMP-gated cation channel alpha-1 |
| **O97564** | 1,7 | Guanine nucleotide-binding protein G(I)/G(S)/G(O) gamma-T2 |
| **P19006** | 1,5 | Haptoglobin |
| **P33703** | 1,9 | Beta-2-glycoprotein 1 |
| **P02677** | 1,5 | Fibrinogen beta chain (Fragment) |
| **P18649** | 1,4 | Apolipoprotein E |
| **P02648** | 2,1 | Apolipoprotein A-I |
| **P81546** | 1,2 | Metalloproteinase inhibitor 1 |
| **F1PTE3** | 2,3 | Ras-related protein Rab-13 |
| **P62999** | 1,1 | Ras-related C3 botulinum toxin substrate 1 |
| **O97555** | 1,2 | Rab GDP dissociation inhibitor alpha |
| **P56595** | 1,6 | Apolipoprotein C-I |
| **P60467** | 1,4 | Protein transport protein Sec61 subunit beta |

| **O18733** | 2,9 | Matrix metalloproteinase-9 |
| --- | --- | --- |
| **P54831** | 1,1 | Transforming growth factor beta-1 |
| **P18067** | 1,1 | Ras-related protein Rab-7a |
| **Q28259** | 1,2 | Glyceraldehyde-3-phosphate dehydrogenase |
| **Q1HE58** | 1,2 | Ras-related protein Rab-27A |
| **Q863A2** | 1,2 | T-box transcription factor TBX2 |
| **P51152** | 1,1 | Ras-related protein Rab-12 (Fragment) |
| **F1PCT7** | 1,5 | Dolichyl-diphosphooligosaccharide--protein glycosyltransferase 2 |
| **O18840** | 1,1 | Actin cytoplasmic 1 |
| **P62822** | 1,2 | Ras-related protein Rab-1A |
| **P60529** | 1,3 | Hemoglobin subunit alpha |
| **P63050** | 1,1 | Ubiquitin-60S ribosomal protein L40 |
| **P80009** | 1,1 | Plasminogen (Fragment) |
| **Q0X0E5** | 1,1 | NADH-cytochrome b5 reductase 3 |
| **P38400** | 1,0 | Guanine nucleotide-binding protein G(i) subunit alpha-2 |
| **Q8WMS6** | 1,0 | Serine/threonine-protein phosphatase PP1-alpha catalytic subunit |
| **P63091** | 1,2 | Guanine nucleotide-binding protein G(s) subunit alpha |
| **Q7YQC6** | 1,1 | Heat shock 70 kDa protein 1 |
| **P79147** | 1,3 | Guanine nucleotide-binding protein G(I)/G(S)/G(T) subunit beta-3 |
| **Q28275** | 1,1 | Fibronectin (Fragment) |
| **P83362** | 1,2 | Signal peptidase complex subunit 1 (Fragments) |
| **P24406** | 1,1 | Transforming protein RhoA |
| **Q50KA9** | 1,1 | Nucleoside diphosphate kinase A |
| **O97492** | 1,0 | Catalase |
| **P61162** | 1,1 | Alpha-centractin |
| **P01784** | 1,1 | Ig heavy chain V region GOM |
| **P49820** | 1,3 | NADH dehydrogenase [ubiquinone] flavoprotein 2 (Fragment) |
| **P61007** | 1,2 | Ras-related protein Rab-8A |
| **P47842** | 1,7 | Solute carrier family 2_ facilitated glucose transporter member 3 |
| **P01874** | 1,1 | Ig chain C region |
| **P00011** | 1,1 | Cytochrome C |
| **P12278** | 19,7 | Apolipoprotein C-II |
| **P28490** | 1,3 | Calreticulin (Fragment) |
| **Q9XSR0** | 1,0 | Interleukin-18 |
| **Q3YIX4** | 1,1 | Phosphatidylethanolamine-binding protein 1 |

| **P86218** | 1,1 | Oligosaccharyltransferase complex subunit OSTC |
| --- | --- | --- |
| **Q28284** | 1,9 | CD44 antigen (Fragment) |
| **Q50KA8** | 1,1 | Nucleoside diphosphate kinase B |
| **Q9TQX6** | 1,0 | Acyl-CoA-binding protein |
| **A0M8V0** | 1,0 | F-actin-capping protein subunit alpha-2 |
| **P24643** | 1,0 | Calnexin |
| **P62490** | 1,0 | Ras-related protein Rab-11A |
| **Q9GL23** | 1,1 | Apoptotic protease-activating factor 1 (Fragment) |
| **P18466** | 1,1 | DLA class I histocompatibility antigen_ A9/A9 alpha chain |
| **Q6TEQ7** | 1,1 | Annexin A2 |
| **E2RQ15** | 1,0 | Ras-related protein Rab-25 |
| **P62872** | 1,0 | Guanine nucleotide-binding protein G(I)/G(S)/G(T) subunit beta-1 |
| **P60952** | 1,0 | Cell division control protein 42 homolog |
| **Q28256** | 1,2 | Platelet glycoprotein Ib alpha chain |
| **P68213** | 1,1 | Fibrinogen alpha chain (Fragment) |
| **Q28294** | 1,0 | Guanine nucleotide-binding protein G(q) subunit alpha |
| **Q28300** | 1,0 | Guanine nucleotide-binding protein G(t) subunit alpha-1 |
| **Q8MJ44** | 1,0 | cAMP-dependent protein kinase catalytic subunit alpha |
| **Q95168** | 1,0 | Tight junction protein ZO-2 |
| **E2RQ08** | 1,0 | Dolichyl-diphosphooligosaccharide--protein glycosyltransferase 1 |
| **P49256** | 1,0 | Vesicular integral-membrane protein VIP36 |
| **E2RE76** | 1,0 | Apolipoprotein A-IV |
| **P19341** | 1,2 | Beta-2-microglobulin (Fragment) |
| **Q28923** | 1,0 | Tyrosine-protein kinase Yes |
| **P33710** | 1,0 | Galanin peptides (Fragment) |
| **Q29536** | 1,0 | Pyruvate kinase PKLR |
| **Q258K2** | 1,0 | Myosin-9 |
| **P01785** | 1,0 | Ig heavy chain V region MOO |
| **Q28250** | 1,0 | Signal peptidase complex subunit 2 |
| **P51147** | 1,0 | Ras-related protein Rab-5C |
| **Q6Q308** | 1,0 | Pleckstrin |
| **P54714** | 1,0 | Triosephosphate isomerase |
| **Q8WNN6** | 1,0 | Superoxide dismutase [Cu-Zn] |
| **P62825** | 1,0 | GTP-binding nuclear protein Ran |
| **P61017** | 1,0 | Ras-related protein Rab-4B |
| **O97556** | 1,0 | Rab GDP dissociation inhibitor beta |
| **Q28288** | 1,0 | Syntaxin-binding protein 2 |
