# Supplementary material for: Platelet proteome changes in dogs with congestive heart failure
Source: BMC Vet Res. 2020 Nov 30;16:466. doi: 10.1186/s12917-020-02692-x (PMC7708215; doi:10.1186/s12917-020-02692-x)
Supplement: Supplementary file 2 — Additional file 2. Protein classification. Protein classification based on the biological process, molecular function, protein class and pathway analysis. [file 12917_2020_2692_MOESM2_ESM.docx]

**Additional file 2:** Protein classifications according to biological processes (file 1), cellular compartment (fig 2), molecular function (fig 3), protein class (fig 4) and pathway analysis (fig 5).


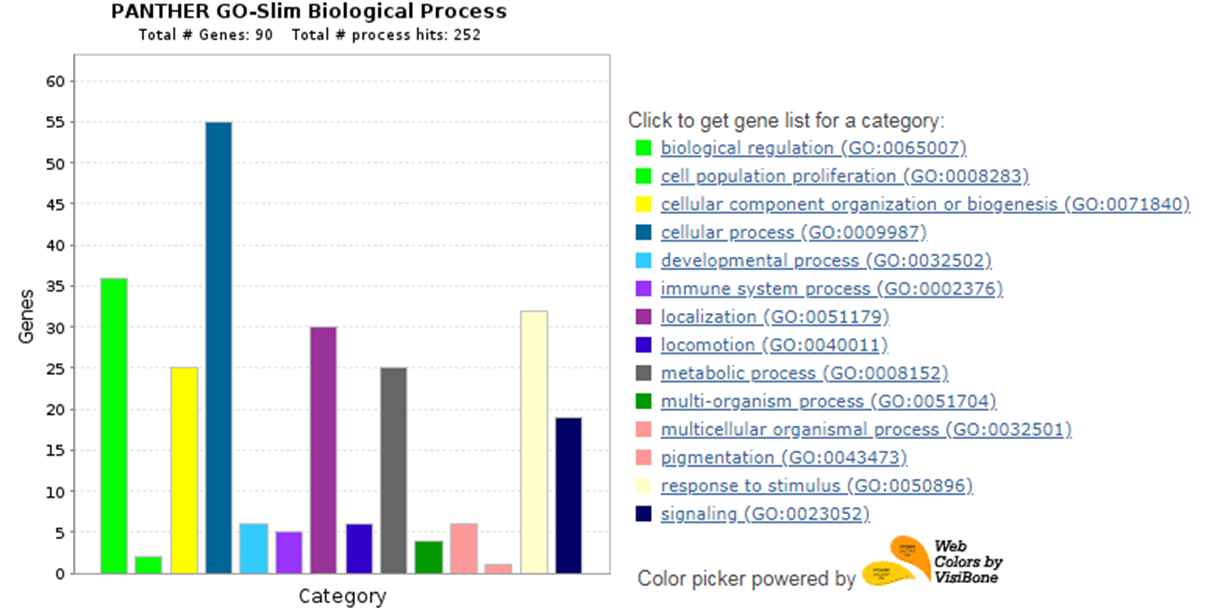


Supplementary Fig 1: Protein classification based on biological processess. Based on the literature the identified 104 proteins could have a role in more than one biological process. Some of the proteins’s biological processess are not known yet.


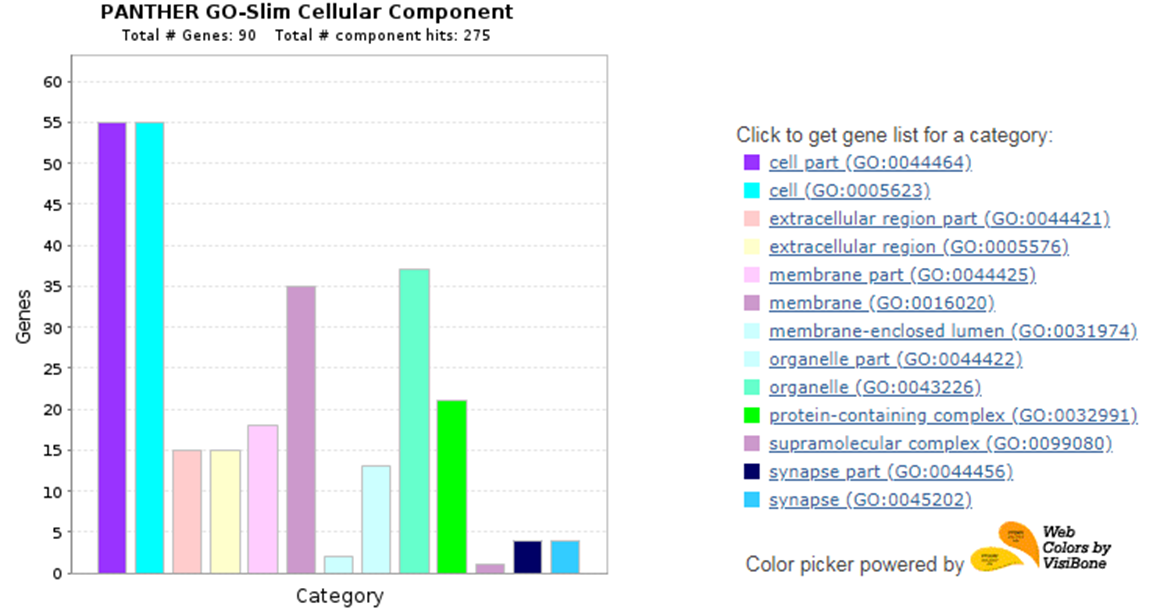


Supplementary Fig 2: The cellular compartment information for the identified proteins.


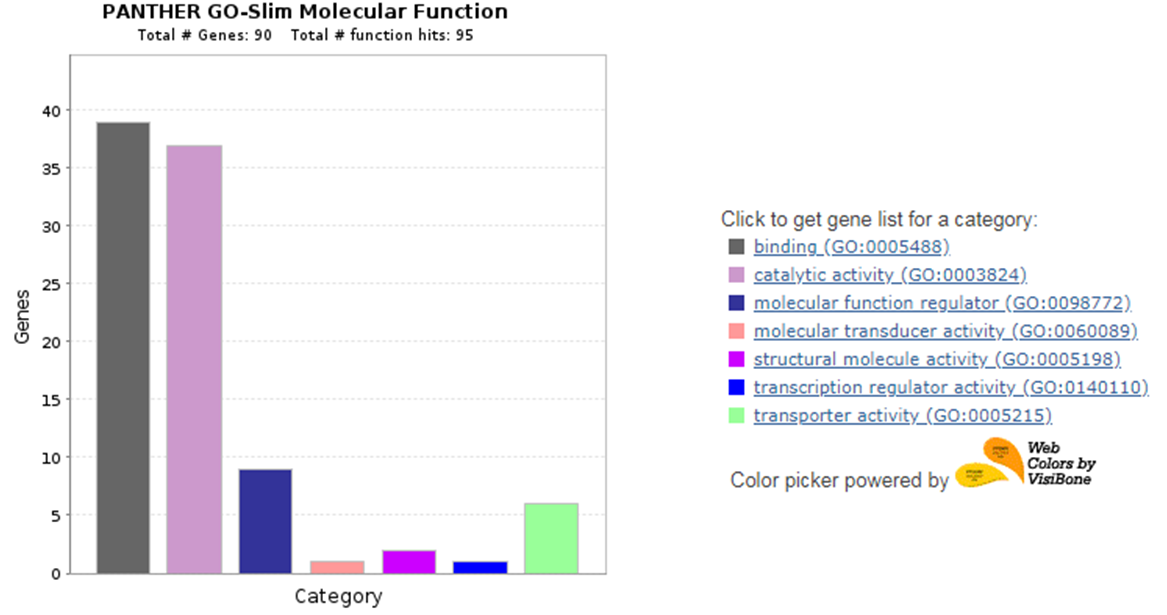


Supplementary Fig 3: Molecular functions carried out by the identified proteins. Only 90 of the 104 identified proteins have known molecular functions.


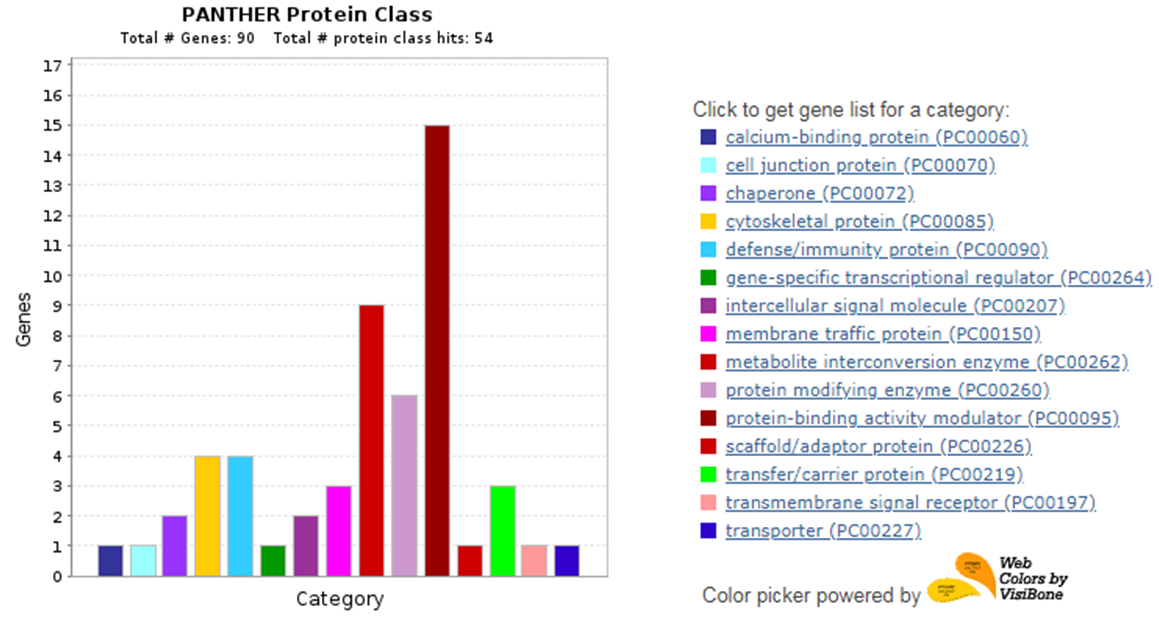


Supplementary Fig 4: Protein class classifications of the identified proteins.


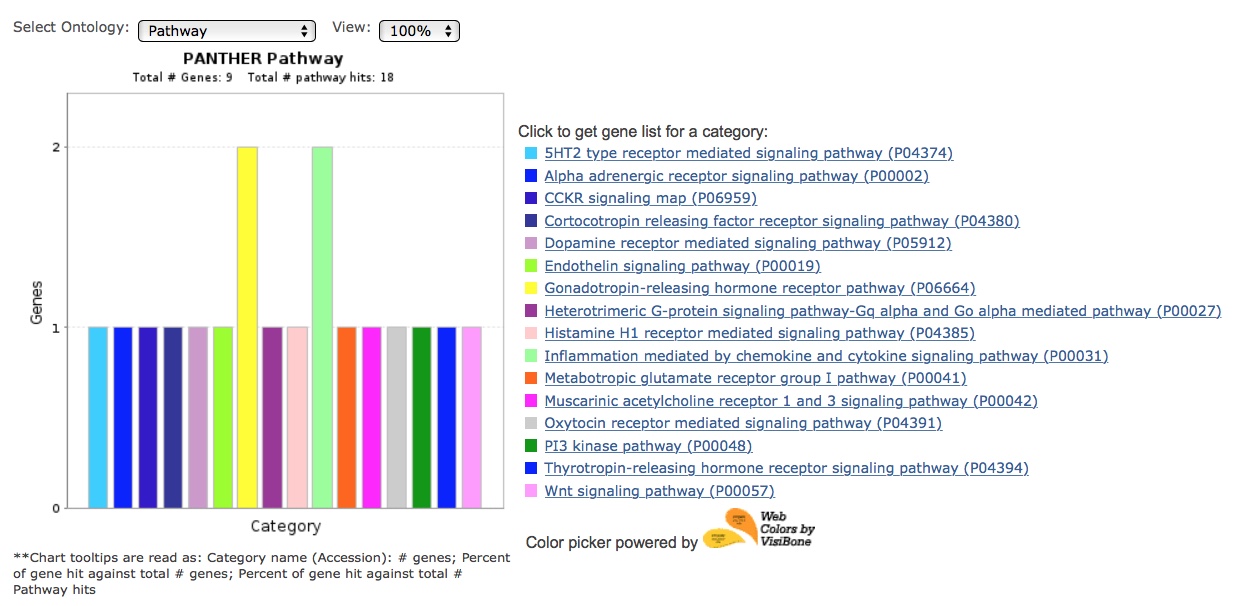


Supplementary Fig 5: Pathway analysis of the identified proteins that were significantly changed.
